# Retraction: Down-regulation of HOXB5 inhibits TGF-β-induced migration and invasion in hepatocellular carcinoma cells *via* inactivation of the PI3K/Akt pathway

**DOI:** 10.1039/d2ra90097a

**Published:** 2022-09-30

**Authors:** Jin-Ping Sun, Quan-Xing Ge, Zheng Ren, Xin-Fang Sun, Shu-Ping Xie

**Affiliations:** Department of Gastroenterology, Huaihe Hospital of Henan University No. 115 Ximen Street, Longting District Kaifeng 475000 China gequanxing_ga@126.com +86-371-23906892 +86-371-23906892

## Abstract

Retraction of ‘Down-regulation of HOXB5 inhibits TGF-β-induced migration and invasion in hepatocellular carcinoma cells *via* inactivation of the PI3K/Akt pathway’ by Jin-Ping Sun *et al.*, *RSC Adv.*, 2018, **8**, 41415–41421, https://doi.org/10.1039/C8RA06860G.

The Royal Society of Chemistry hereby wholly retracts this *RSC Advances* article due to concerns with the reliability of the data.

The western blots in Fig. 1B, 1D, 2A, 2B and 4A have been over-contrasted to the point where the background has almost been erased.

In Fig. 2C, the ‘shControl/−’ and ‘shHOXB5/+’ panels are rotated versions of the same image. Some additional features can be observed in the ‘shHOXB5’ image, although the majority of the features overlap between the two images.

In Fig. 2D, the ‘shControl/−’ and ‘shHOXB5/−’ panels contain many duplicating features. Some additional features can be observed in the ‘shControl’ image, although the majority of the features overlap between the two images.

Two of the images in the ‘shHOXB5 + TGF-β’ panel in Fig. 3A are identical.

In Fig. 4B, the ‘shControl/−/−’ and ‘shHOXB5/+/−’ panels are rotated versions of the same image. Some additional features can be observed in the ‘shHOXB5/+/−’ image, although the majority of the features overlap between the two images.

The authors were asked to provide the raw data for this article, but did not respond. Given the significance of the concerns about the validity of the data, and the lack of raw data, the findings presented in this article are not reliable.

The authors were informed but have not responded to any correspondence regarding the retraction.

Signed: Laura Fisher, Executive Editor, *RSC Advances*

Date: 18^th^ August 2022

## Supplementary Material

